# Valorization of Raw Wool Waste as a Sustainable and Biodegradable Alternative Infill for Artificial Turf Systems

**DOI:** 10.3390/ma19194154

**Published:** 2026-09-29

**Authors:** Luisa Coderch, Meritxell Martí, Cristina Alonso, Javier Batlle, Manuel-José Lis, Gabriela Mijas

**Affiliations:** 1 Institute of Advance Chemistry of Catalonia (IQAC-CSIC), Jordi Girona 18-26, 08034 Barcelona, Spain; luisa.coderch@iqac.csic.es (L.C.); cristina.alonso@iqac.csic.es (C.A.); 2 Department of Materials Science and Engineering (CEM), Institute of Textile Research and Industrial Cooperation of Terrassa (INTEXTER), Universitat Politècnica de Catalunya·BarcelonaTech (UPC), C. Colom, 11, 08222 Terrassa, Spain; meritxell.marti.gelabert@upc.edu; 3 Infortecnia, Francesc Carbonell 50, 08034 Barcelona, Spain; javierbatlle54@gmail.com; 4 Department of Chemical Engineering (EQ), Institute of Textile Research and Industrial Cooperation of Terrassa (INTEXTER), Universitat Politècnica de Catalunya·BarcelonaTech (UPC), C. Colom, 1, 08222 Terrassa, Spain; manuel-jose.lis@upc.edu

**Keywords:** wool, styrene-butadiene rubber (SBR), artificial grass, turf, pellets, advanced artificial athlete tester (AAA), circular economy, waste valorization

## Abstract

This study presents a sustainable circular economy approach to valorize raw wool waste by developing engineered, biodegradable bio-pellets as a functional replacement for styrene-butadiene rubber (SBR) infills in synthetic turf systems. To overcome physical limitations and address upcoming regulatory restrictions on microplastics (Regulation (EU) 2023/2055), raw wool was processed into dense pellets through controlled water inclusion and compounding with density-increasing mineral additives (Dolomite and Barite). Thermogravimetric Analysis (TGA) was utilized to precisely quantify the inorganic fractions and thermal stability of the resulting formulations. The physical, mechanical, and biomechanical behavior of the wool-based composites was comprehensively evaluated, focusing on Shock Absorption (SA), Vertical Deformation (VD), and Energy Restitution (ER) across different elastic subbases. Results confirm that the developed bio-pellets fit within the stablished ranges of international mechanical standards (EN 15330-1, FIFA, and World Rugby). Furthermore, moisture-induced swelling permanently enhances damping performance and prevents particle displacement, while the intrinsic hydrophilic nature of the keratin matrix could provide hydro-thermal stabilization and reduce electrostatic charges compared to conventional SBR crumbs. By leveraging existing fertilizer processing infrastructure, this work demonstrates a scalable route to transform a problematic agricultural waste into a high-performance, non-toxic, and eco-friendly material for advanced engineering applications.

## 1. Introduction

Artificial turf infill is a granular material that covers the base of the synthetic fibers, mimicking the role of soil in natural grass systems. Its primary functions include maintaining fiber uprightness, providing cushioning and impact absorption, securing the turf carpet in place, and mitigating surface overheating [1,2,3].

Styrene-butadiene rubber (SBR) particles, derived from recycled waste tires, remain one of the most widely used materials for the upper infill layers due to their high availability, affordability, and excellent cushioning properties [4]. However, critical concerns have emerged regarding the environmental and health impacts of crumb rubber. These granules contain potentially hazardous substances, including polycyclic aromatic hydrocarbons (PAHs), per- and polyfluoroalkyl substances (PFAS), heavy metals, phthalates, and both volatile and semi-volatile organic compounds (VOCs and SVOCs) [5,6]. Furthermore, their migration into surrounding soil and water ecosystems significantly contributes to microplastic pollution [7]. These environmental and toxicological drawbacks have accelerated the market growth for alternative infills, ranging from synthetic to mineral and plant-based materials. Currently, a definitive hierarchy among alternative fillers is lacking. While untreated plant-based materials generally present the lowest chemical toxicity, they introduce biogenic risks such as mold proliferation, respirable fibers, and allergen exposure [8]. Consequently, a critical need persists for safe, eco-friendly infill materials that deliver robust mechanical performance without compromising economic viability or availability.

From the 17th century to the first half of the 20th century, the wool industry was the one of the leaders in textile industry; however, demand has since been severely compromised by competition from synthetic fibers and shifting consumer preferences toward lighter, casual apparel [9]. Today, significant volumes of raw shorn wool remain unmarketed due to textile quality deficiencies, a lack of buyers, or insufficient localized processing infrastructure [10]. Consequently, annual sheep shearing, a mandatory practice for animal welfare, regardless of whether livestock is raised for meat or milk, has transitioned from a profitable revenue stream into a financial and logistical burden [11]. While a negligible fraction satisfies artisanal standards, the vast majority of greasy wool is stockpiled indefinitely or discarded in natural environments [12]. To address this critical disconnect between livestock production and sustainable land management, the global pursuit of sustainability has prompted the reconsideration of raw wool residues as a renewable, biodegradable resource for value-added materials [9,13].

In this context, understanding the baseline chemical and physical composition of raw, greasy wool is essential to justify its performance in existing industrial applications and to unlock its potential for novel engineering solutions. Although fleece composition varies according to breed, husbandry practices, environmental conditions and sampling location, greasy Merino wool typically contains approximately 45–60 wt% clean wool fibre, 15–16 wt% wool grease, 15 wt% mineral dirt, 5 wt% suint and smaller proportions of vegetable and proteinaceous contaminants [14,15,16]. The principal solid component is the keratin-based wool fiber, whereas wool grease, commonly referred to as crude lanolin, is a complex mixture dominated by high-molecular-weight esters formed from long-chain fatty acids and sterol or aliphatic alcohols [17]. Suint consists of dried, water-soluble perspiration residues and contains predominantly potassium salts of organic and fatty acids [16,18]. From an end-of-life perspective, wool has a high nitrogen content and consequently a relatively narrow carbon-to-nitrogen ratio compared with most lignocellulosic biomasses. This composition supports microbial degradation and nutrient release under biologically active soil or composting conditions [19]. Nevertheless, because keratin is a chemically resistant, disulfide-crosslinked protein, degradation rates depend on environmental conditions and should be described as gradual biodegradation and nutrient cycling [20]. Unlike persistent synthetic polymer, wool-based materials can ultimately be mineralized by microorganisms without generating persistent microplastics [21].

Diverse non-apparel applications have consequently emerged, ranging from building insulation, oil spill absorbents, and tissue engineering scaffolds to advanced carbon-based nanofibers [22,23,24,25]. Furthermore, raw wool is widely utilized as an organic fertilizer; its inherent thermal insulation and moisture-retention properties protect crops from ambient fluctuations, while its slow biodegradation kinetics ensure a sustained release of essential nutrients [26,27]. Ultimately, exploring such unconventional applications is crucial to sustaining sheep farming, a vital, traditional socio-economic activity in many global rural regions where alternative revenue models are scarce.

In this context, an innovative, biodegradable, and non-toxic natural infill material could also be considered for use in artificial turf systems. Wool possesses several inherent properties that make it a promising sustainable and functional alternative to conventional polymeric infills. Firstly, wool is a highly hygroscopic fiber capable of absorbing and desorbing substantial amounts of moisture [28,29]. This moisture-buffering behavior contributes to its thermoregulatory properties, since moisture desorption and evaporation require heat and may therefore provide a cooling effect [29,30]. Secondly, wool exhibits high elasticity and compressional resilience as a result of its natural crimp and keratin-based hierarchical structure, enabling the fibers to deform under load and subsequently recover their original configuration [29,31,32]. These characteristics make wool potentially advantageous for cushioning and impact-energy dissipation applications. Thirdly, wool is a natural, renewable, and biodegradable keratin-based fiber. Wool fibers have been shown to biodegrade under both aerobic composting and marine conditions, whereas synthetic materials such as polyester, nylon, and polypropylene exhibit substantially lower biodegradation under comparable conditions [19,21]. Consequently, the use of wool could reduce the potential for persistent synthetic microplastic accumulation associated with conventional polymeric materials [21]. Finally, wool possesses inherent resistance to ignition, with untreated wool typically exhibiting a limiting oxygen index (LOI) of approximately 25% [29,33]. This intrinsic low flammability may reduce the need for additional flame-retardant treatments and represents a potential advantage for applications subject to fire-safety requirements.

The main objective of this work is to evaluate raw wool as a novel, biodegradable, and non-toxic natural alternative infill for artificial turf systems. This study investigates how the unique macromolecular structure and high keratin content of raw wool provide elastomeric properties comparable to traditional rubber (SBR), while exploiting its inherent moisture absorption to enhance player comfort and reduce surface static electricity. Ultimately, this research aims to validate a sustainable, closed-loop solution that mitigates the environmental and health hazards associated with synthetic infills, thereby supporting the economic viability of traditional sheep farming.

## 2. Materials and Methods

### 2.1. Raw Material and Pre-Treatment

Raw Spanish wool provided by Pius Montanyà Pineda (Barcelona, Spain) was used to manufacture the infill pellets. The wool fractions selected were obtained from fleece bypass streams rejected during the shearing campaign due to their low textile quality. Prior to pelletization, the raw wool underwent a size reduction process using a commercial cutter (Elf HL20, Elf Systems, Prague, Czech Republic) to grind the fibers into a regular, repeatable size range, ensuring a consistent texture for subsequent processing.

### 2.2. Pelletization Process

**Figure 1:**
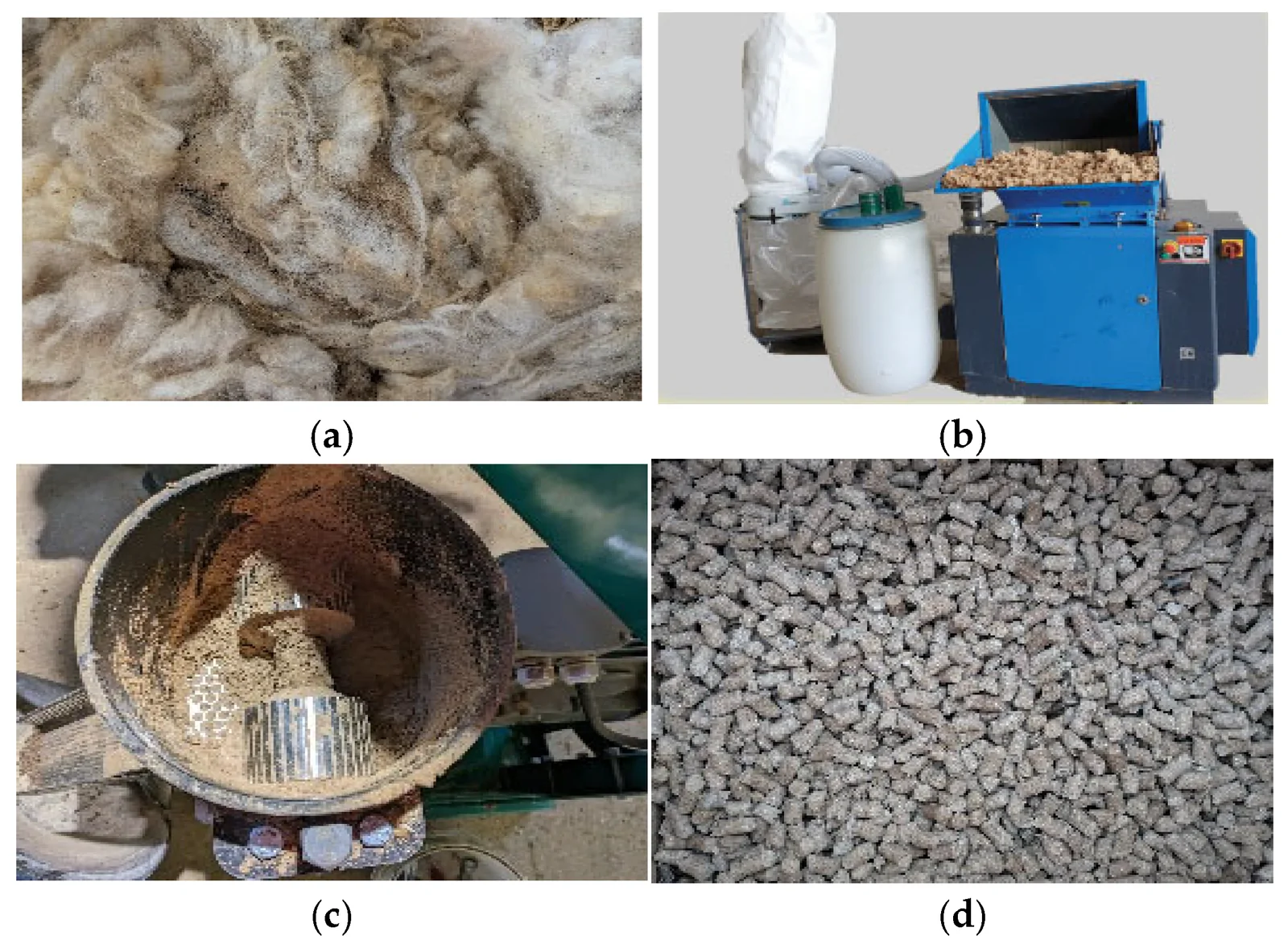
Production of wool pellets for artificial turn infill: (**a**) raw wool; (**b**) size reduction; (**c**) milling; (**d**) wool pellets.

The pre-treated wool was compacted into pellets using an Elf HB200 die pellet mill equipment (Elf Systems, Prague, Czech Republic). The standard pellet diameter was set at 6.0 mm, though specific batches were reduced to 4.5 mm and 3.0 mm to evaluate size suitability. Due to the high natural lanolin and moisture content of the raw wool, the fibers successfully bonded under pressure without requiring external binder additives; the grease acted as a natural processing aid to ensure structural integrity. Pelletization trials were conducted at both Elf Systems and the Fundación Grazalema Regenerativa (Cadiz, Spain) (Figure 1).

### 2.3. Density Optimization and Sanitization

In order to match the technical requirements of artificial turf, the baseline density of the pure wool pellets was increased by incorporating high-density inorganic mineral salts. The compounding additives evaluated included barium sulfate (barite) (98% extra pure, density 4.5 g/cm^3^, Thermofisher Scientific, Geel, Belgium) and dolomite (size 100 µm, bulk density 1.5 g/cm^3^, Mineravi, Alfaro, Spain). Finally, to ensure hygiene and biological safety for sports surfaces, the manufactured pellets were sanitized via thermal treatment in a controlled Pol-Eko SLW1000 oven (Wodzisław Śląski, Poland) at 100 °C for at least 125 min under an absolute pressure of 1 bar, complying with transformation Method 3 of Commission Regulation (EU) No. 142/2011, Annex IV [34] for sterilization. Furthermore, as required by the same regulation, periodic microbiological analyses were conducted to ensure that the treatment achieves the required biosecurity standards.

### 2.4. Material Characterization

To determine bulk density, the wool pellet samples were conditioned in a climate-controlled room at 25 °C and 50% relative humidity (RH) for at least 48 h before being weighed within a fixed volume of 25 mL.

Thermogravimetric Analysis (TGA) was subsequently performed on the conditioned samples using a Discovery SDT 650 analyzer (TA Instruments, New Castle, DE, USA). Samples of approximately 5 mg were placed in open aluminum pans and heated from 25 °C to 850 °C at a constant heating rate of 10 °C/min under a nitrogen (N_2_) flux of 60 mL/min to determine the mass fractions of moisture, organic matter, and inorganic ash. To ensure complete combustion of resilient organic residues (such as acid carbon residue or carbon black) that may persist above 550 °C under inert conditions, the purging atmosphere was automatically switched to oxygen (O_2_) at 600 °C.

### 2.5. Small-Scale Experimental Setup and Sample Preparation

**Figure 2:**
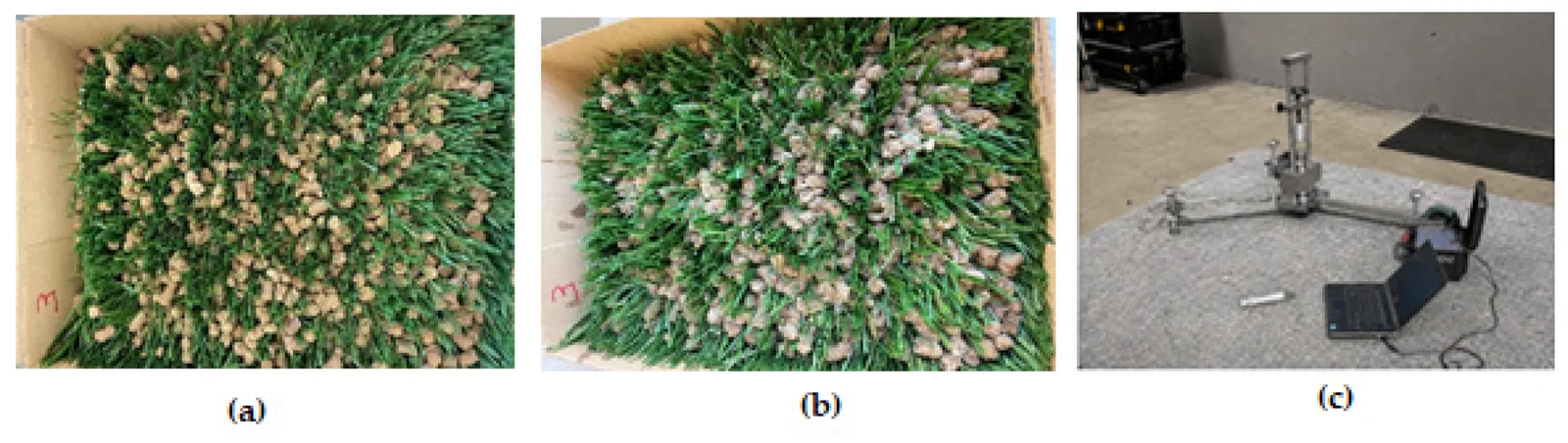
(**a**) Wool pellets in artificial turf specimen, (**b**) Wool pellets in artificial turf specimen with 600 mL of water (~9.8 L/m^2^) and (**c**) Advanced artificial athlete tester (AAA).

To evaluate the mechanical performance of the alternative infills on a small scale, artificial turf specimens were prepared using standard DIN A4 dimensions (0.0609 m^2^) as seen in Figure 2a. The control system simulated a standard football pitch configuration, consisting of 60 mm polypropylene a fiber carpet (RGF XM7 60-10.5, Fieldturf Poligras, Sant Cugat, Spain) infilled with 1.5 kg of silica sand (≈25 kg/m^2^, RP-0508, Euroarce Mining, Zaragoza, Spain) and a proportional rubber infill mass of 1.0 kg of styrene-butadiene rubber (SBR), equivalent to a usual dosage full-scale application of 16 kg/m^2^.

In parallel, experimental specimens were prepared by substituting the SBR with the manufactured wool pellets. To optimize the system’s performance, the following experimental variables were systematically evaluated:

- Pellet size: Assessment of different pellet diameters (3.0 mm, 4.5 mm, and 6.0 mm).
- Pellet density: Evaluation of pellets compounded with varying concentrations of high-density mineral additives.
- Infill dosage: Variation in wool pellet mass ranging from 50 to 200 g per specimen (equivalent to approximately 1.0 to 3.0 kg/m^2^).
- Moisture content: Addition of water volumes ranging from 300 to 600 mL per specimen (equivalent to 5.0 to 10.0 L/m^2^) to simulate diverse environmental and weather conditions (Figure 2b).

Selected specimens underwent comprehensive physical, chemical, and mechanical characterization. Mechanical tests were conducted by placing the simulated turf samples directly onto a rigid concrete floor or over a shock-absorbing elastic layer (ProPlay Sport, Schmitz Foam Products, Roermond, The Netherlands), which is commercially utilized to compensate for alternative infills with lower intrinsic elasticity than conventional rubber.

### 2.6. Mechanical Performance and Standard Requirements

Shock absorption (SA), vertical deformation (VD), and energy restitution (ER) represent the three primary attributes governing impact attenuation and safety in artificial turf systems. These properties are strictly regulated by international sports governing bodies (e.g., FIFA and World Rugby) and general standards such as EN 15330-1.

To reproduce the dynamic impact of an athlete’s heel strike during running, standard drop-test methods were employed using the “Artificial Athlete” (AA) and “Advanced Artificial Athlete” (AAA) tester (Figure 2c) (Deltec Equipment, Duiven, The Netherlands). The apparatus comprises a mechanical release mechanism, a falling mass, a calibrated linear spring, an impactor, and an accelerometer. The recorded acceleration-time data allows for the direct calculation of surface SA, VD, and ER. These tests were conducted using specialized equipment and under the technical supervision of Fieldturf Poligras.

The apparatus configurations and experimental methodologies comply with established standards, including ASTM F3189-17, FIFA Test Methods (2015, 2019), CEN/TS 16717, and EN 15330-1, as well as literature guidelines [35]. The specific performance requirements dictated by different standard bodies are summarized in Table 1. It should be noted, however, that the measurements were carried out on small DIN A4 samples. Therefore, based on the values obtained under the stated laboratory protocol, it can only be determined whether they fall within the selected standard ranges.

## 3. Results

### 3.1. Pellet Production Mechanics and Density Optimization

To develop a functional natural infill tailored for soccer and rugby pitches, surfaces strictly requiring elastic, shock-attenuating media, the raw Spanish wool was processed into small, high-density granules. The pelletization mechanism relied on a mechanical die-and-roller matrix configuration. The rollers exerted a continuous compressive force, pushing the shredded biomass into the tapered die orifices. This localized restriction induced simultaneous friction, shear, and thermal energy, effectively fusing the keratinous fibers into cohesive, solid cylindrical pellets without the addition of synthetic binders.

The process efficiency required a strict optimization of the feedstock geometry and moisture content. The raw wool was initially comminuted to a repeatable size range matching or falling below the die diameter. While standard industrial pellet mills typically utilize 6.0 mm dies, this study evaluated reduced orifices down to 3.0 mm to favor turf penetration. Furthermore, feedstock moisture was maintained within the critical 10–20% process window; at an ambient 50% RH, the inherent moisture of the raw wool (10–12%) provided the necessary back-pressure within the die, eliminating the need for energy-intensive pre-drying or water-conditioning stages.

To overcome the naturally low bulk density of pure wool, high-density mineral compounds (Dolomite and Barite) were incorporated into the matrix at formulations of 25 wt% and 50 wt% (relative to dry wool weight). The physical properties and dimensional variations of the resulting experimental batches are listed in Table 2, with all measurements performed in triplicate.

The experimental data reveals a profound impact of both mechanical compaction and mineral compounding on the volumetric properties of the material. Uncompressed clean wool (Sample 1) exhibits an extremely low bulk density of 0.03 g/cm^3^, which would cause the material to float or be easily displaced by wind on an athletic field. Mechanical pelletization alone (Sample 2) successfully increases this baseline value to 0.39 g/cm^3^ due to the forced elimination of macromolecular void spaces.

The integration of mineral fillers further optimizes these physical traits. Incorporating 25 wt% and 50 wt% of Dolomite (Samples 3 and 4) increased the density to 0.67 g/cm^3^ and 0.90 g/cm^3^, respectively. The highest volumetric weight was achieved using 50 wt% Barite at a 6.0 mm diameter (Sample 5), reaching 0.93 g/cm^3^, which represents a 31-fold density increase compared to the raw fleece.

Interestingly, when altering the spatial dimensions of the 50 wt% barite pellets from 6.0 mm down to 4.5 mm (Sample 6) and 3.0 mm (Sample 7), a minor density reduction was noted (decreasing from 0.93 to 0.87 g/cm^3^). This trend is attributed to altered flow dynamics and shear stresses within narrower die channels, which slightly limits maximum compaction. Nevertheless, all mineral-compounded pellets (Samples 3–7) achieved values near or exceeding 0.70 g/cm^3^, confirming that both size and density can be precisely modulated during manufacturing. Minimizing pellet diameter while maintaining a high volumetric density is highly desirable for sports surfaces, as it promotes gravity-driven particle migration and stable penetration into the deep thatch of synthetic turf fibers.

### 3.2. Thermogravimetric Analysis (TGA)

Thermogravimetric Analysis (TGA) was utilized to quantitatively determine the compositional mass fractions of the wool samples and the manufactured pellets. By monitoring sample mass variations as a function of temperature under a controlled thermal program, this technique enables the precise identification of volatile and non-volatile components up to a final temperature of 850 °C.

The initial mass loss step corresponds to the evaporation of desorption-bound water, followed by a primary degradation stage driving the pyrolysis of organic macromolecular compounds, specifically the keratinous wool protein and natural wool grease (lanolin). Under standard inert conditions, certain resilient organic fractions, such as acid carbon residues and carbon black, can persist at temperatures above 550 °C. To ensure complete combustion and accurate quantification of the true inorganic fraction, the purging atmosphere was automatically switched from nitrogen (N_2_) to oxygen (O_2_) at 600 °C. The resulting mass distribution parameters (water: room temperature-150 °C, organic fraction: 150–600 °C, inorganic fraction: 600–850 °C) for the different formulations are summarized in Table 3, with all measurements performed in triplicate.

The TGA thermograms indicate that when conditioned at 50% RH, clean wool exhibits a moisture content of 9.2%, which is highly comparable to the 9.4% observed in the raw wool pellets. Notably, clean wool displays the highest organic matter content at nearly 90%, leaving a minor inorganic ash residue of only 2.0%. In contrast, the unwashed raw wool pellets yield a significantly higher inorganic fraction of 14.0%. This sharp baseline increase directly accounts for the presence of environmental dirt, sand, and mineral contaminants embedded within the unscoured fleece during the shearing campaign.

The evaluation of the mineral-compounded specimens (Samples 3–5) indicates a predictable, concentration-dependent increase in the inorganic fraction. This enrichment in stable mineral phases consequently dilutes the total organic matrix percentage. Furthermore, a systematic decrease in the overall water content is observed, dropping to 4.3% in the 50% Dolomite formulation. This behavior is physically governed by the non-hygroscopic nature of the inorganic salts; since moisture retention is exclusively driven by the hydrophilic groups within the wool’s protein fraction, increasing the mineral loading effectively reduces the hydroactive polymer volume per unit mass of the composite pellet.

### 3.3. Mechanical Performance on Rigid Substrates (Concrete Subbase)

Understanding the compressive and shear behavior of the fill requires an assessment of the overall mechanical performance of the artificial turf [3]. Accordingly, the independent shock-damping capacity of the wool matrix was initially evaluated under a worst-case scenario by placing the turf specimens directly onto a rigid concrete floor. A bare concrete slab lacks compliance, exhibiting a baseline shock absorption (SA) of 0%, a vertical deformation (VD) of 0 mm, and an energy restitution (ER) of 100%. Therefore, the entire burden of impact attenuation, energy dissipation, and player safety rests solely on the turf carpet and the infill design. The mechanical response as a function of pellet dosage, mineral compounding, and moisture pre-treatment, assessed in triplicate, is summarized in Table 4.

#### 3.3.1. Analysis of Shock Absorption (SA)

The control specimen containing only silica sand (Sample A) demonstrated an insufficient SA of 22.2%, failing to meet the minimum 55% threshold mandated by EN 15330-1 and FIFA standards. The introduction of 100 g of raw wool pellets (Sample B) significantly enhanced impact damping to 31.2%, a value that further escalated to 44.1% when doubling the infill mass to 200 g (Sample D). This mass-dependent improvement confirms the high intrinsic elasticity and rubber-like behavior of the compacted keratinous fibers.

Crucially, a permanent structural modification was observed following the water pre-treatment. Although all mechanical measurements were executed after the specimens had completely dried, the wet-and-dry cycle induced a substantial increase in SA, reaching 37.5% for the 100 g dosage (Sample C) and a maximum of 47.1% for the 200 g dosage (Sample E). This phenomenon is driven by the hygroscopic history of wool: the localized absorption of water swells the protein chains, permanently softening the pellet architecture and increasing micro-porosity upon drying, which optimizes pneumatic and mechanical energy dissipation during impact. It should be noted that these results remain unchanged after successive wetting and drying cycles, as the pellets disintegrate during the initial stage, allowing the wool to form a homogeneous surface that contributes to maintaining the mechanical performance. Specimens compounded with Dolomite (Samples F and G) also maintained elevated SA levels (≈39.4%), proving that mineral loading does not compromise shock attenuation. However, despite these major improvements, none of the configurations reached the 55% compliance limit when tested over bare concrete, highlighting the need for an underlying elastic shockpad.

#### 3.3.2. Analysis of Vertical Deformation (VD) and Energy Restitution (ER)

Unlike shock absorption, the vertical deformation and energy restitution metrics achieved full regulatory compliance even in the absence of an elastic subbase. While the sand control (Sample A) fell short with a restrictive VD of 2.5 mm, the addition of wool pellets successfully shifted the deformation into compliant boundaries. The 100 g hydrologically treated specimen (Sample C) and both 200 g configurations (Samples D and E) yielded VD values between 4.4 mm and 5.9 mm. These data points sit comfortably within the 4–9 mm range required by EN 15330-1 and the 4–11 mm spectrum enforced by FIFA.

Concurrently, the turf system must minimize excessive energy restitution to protect athletes from joint strain and unnatural surface rebound. The sand control (Sample A) exhibited an undesirable ER of 62.2%. The integration of wool pellets effectively suppressed this rebound effect through structural friction and viscoelastic damping. Increasing the wool pellet mass and applying the moisture pre-treatment reduced the ER to a minimum of 31.2% (Sample E). Notably, specimens C through G fell squarely within the strict 22–48% energy restitution window mandated by World Rugby. These findings demonstrate that from a kinematic standpoint, a top-layer infill composed of raw wool pellets delivers the necessary surface compliance (VD) and non-injurious energy return (ER) required for elite contact sports, even under rigid subbase conditions.

### 3.4. Mechanical Performance on Elastic Substrates (Shockpad System)

To address the shock absorption deficits observed under rigid subbase conditions and to simulate elite sports facility architecture, the experimental synthetic turf specimens were evaluated over a 23 mm prefabricated closed-cell polyolefin elastic base (ProPlay Sport, Schmitz Foam Products). This system represents the standard engineering configuration for alternative, bio-based infills that possess lower baseline bulk elasticity than traditional vulcanized rubber granulates. The mechanical parameters recorded for the turf system supported by the shockpad are detailed in Table 5. All tests were performed in triplicate.

#### 3.4.1. Regulatory Compliance and Synergy with Elastic Subbases

The integration of the 23 mm elastic base dramatically shifted the mechanical response of the entire pavement system, bringing every single experimental configuration into full compliance with international sports regulations. The control specimen (Sample A, sand-only) achieved a baseline SA of 60.8%, a VD of 7.5 mm, and an ER of 36.5%, which already satisfies the intermediate criteria of EN 15330-1 and FIFA. Furthermore, according to a previous report issued by an accredited laboratory (Labosport, Le Mans, France), a sample containing 16 kg/m^2^ of SBR without an elastic base and another containing 9 kg/m^2^ of SBR with ProPlay showed the following results: an SA of 64%, a VD of 9.1 mm, and an ER of 38%. The introduction of the raw wool pellets introduced a distinct performance-enhancing trend.

As observed in the rigid subbase trials, adding the wool pellets systematically elevated both shock absorption and vertical deformation while concurrently suppressing energy restitution. Pure raw wool pellets at a 100 g dosage (Sample B) increased SA to 62.2%, while the 200 g dosage (Sample D) pushed it to 63.1%. When incorporating the mineral fillers, dolomite and barite (Samples F and G), the system maintained excellent mechanical compliance, yielding SA values up to 66.6% and VD values up to 9.0 mm, comparable to those obtained with SBR. Only some of the results obtained for Sample B were not significantly different from those obtained for Sample A. These results confirm that compounding raw wool with high-density mineral salts to improve particle weight and prevent field migration does not compromise the collaborative macro-elasticity of the turf-shockpad matrix.

#### 3.4.2. Morphological Transformation and Hydrological Dispersion Mechanism

The most remarkable mechanical enhancement was consistently triggered by the water pre-treatment. Sample E (200 g of wool pellets with 600 mL of water) achieved the absolute highest impact attenuation performance of the entire study, recording a maximum SA of 66.7%, an optimized VD of 8.9 mm, and a highly protective ER of 29.3%. This prominent trend is governed by a dual physical and morphological mechanism activated during the wet-and-dry cycle. The disintegration of the granules due to moisture occurs only during the initial contact with water, as observed during the water pre-treatment (Figure 2b). Thereafter, a homogeneous and continuous protein fiber structure is formed and remains stable upon subsequent exposure to water. Tests under real-world conditions are currently underway to assess its long-term behavior and extrapolate its performance over the expected lifespan of the artificial turf.

First, at a macromolecular level, the influx of water molecules induces extensive swelling of the amorphous regions within the keratin protein chains. This hydration disrupts secondary hydrogen bonds, temporarily increasing the free volume and structural flexibility of the fibers. Second, at a macroscopic level, the hydrological exposure triggers the structural de-agglomeration of the compressed pellets. Because raw wool pellets are manufactured utilizing the natural suint (water-soluble potassium salts of fatty acids) and lanolin as the sole binding mechanisms under pressure, the application of water dissolves the water-soluble suint fraction.

Consequently, the compressed granules undergo controlled disintegration, allowing the highly elastic, curled wool fibers to release and distribute isotropically within the silica sand thatch. This transformation converts localized, rigid pellet geometries into a highly uniform, homogeneous, and continuous fibro-protein mat across the turf surface. This continuous wool matrix optimizes pneumatic dampening and structural friction during the heel-strike impact of the Artificial Athlete apparatus, maximizing mechanical energy dissipation. These findings corroborate that raw wool infills offer a highly adaptive, self-homogenizing performance profile that successfully fulfills the strict biomechanical and safety criteria mandated by FIFA and World Rugby.

## 4. Discussion

The development of raw wool pellets as a functional infill addresses a critical regulatory vacuum in the sports surface industry. The European Union’s upcoming 2031 ban on intentionally added microplastics will completely prohibit the use of traditional styrene-butadiene rubber (SBR) crumb infills across all synthetic turf applications [36,37]. In this context, transitioning to a biodegradable and renewable resource directly aligns with the toxic-free environment goals of the European Green Deal [38].

From a circular economy perspective, this strategy introduces a highly underutilized agricultural by-product into a high-value industrial chain. Currently, raw wool fractions unsuitable for high-quality textile processing possess zero commercial value, forcing sheep farmers to incur disposal costs or resort to environmentally damaging management practices, such as open-field stockpiling or incineration [12,39]. Crucially, the manufacturing route proposed herein utilizes unwashed raw wool, bypassing the energy-intensive and highly polluting chemical scouring processes typical of the textile sector [40]. This unconventional valorization pathway transforms a financial liability for rural communities into an economic asset, supporting the financial viability of extensive sheep farming and fostering new bio-based industrial activities in rural areas.

A major advantage of the proposed infill is its rapid industrial scalability. Raw wool pellets are already manufactured globally on a commercial scale for use as organic fertilizers due to their rich nitrogen and sulfur content [41,42]. Because the pelletization mechanics, machinery, and thermodynamic processing windows have already been optimized within the agricultural sector, scaling up production for sports field applications requires only minor compositional and geometric adjustments (such as the mineral compounding validated in Section 3.1). This eliminates the capital-intensive infrastructure barriers typically associated with the commercialization of novel bio-composites.

The mechanical data compiled in Section 3.3 and Section 3.4 demonstrate that wool-based infills successfully would meet the strict biomechanical safety boundaries enforced by FIFA and World Rugby. It is expected that the limitations associated with the small sample size used in these studies may also affect the performance of the system at full scale. Beyond structural compliance, the intrinsic thermodynamic and hygroscopic traits of the raw wool matrix offer superior playing comfort compared to synthetic SBR elastomers.

A persistent operational drawback of SBR fields is the accumulation of static electricity and excessive heat retention, particularly in regions subject to high solar radiation. Although the electrostatic behavior of the wool-filled samples has not yet been assessed, the high moisture absorption capacity of the keratin fibers, which maintain a baseline moisture content of approximately 10%, may contribute to the dissipation of accumulated electrostatic charge [43].

Furthermore, this hydro-active behavior yields unprecedented water conservation benefits. Traditional SBR pitches require intensive irrigation before and during matches to mitigate electrostatic buildup and lower surface temperatures [44]. In arid or water-scarce regions, this operational requirement exacerbates local water stress. Although the mechanical performance improves after pre-wetting the wool pellets, this effect appears to persist over time, and no additional wetting is required to maintain the observed properties. The moisture retention of the wool matrix (10–12%) is projected to reduce pitch irrigation demands compared to SBR crumbs, minimizing municipal water consumption and lowering the operational carbon footprint of sports complexes.

While the long-term durability and in-field degradation dynamics of wool infills remain under active investigation, their environmental degradation pathways present a highly favorable profile. Upon exposure to ambient humidity, the pellets undergo a controlled, moisture-driven de-agglomeration. As the granules absorb water, they swell, soften, and break down into a uniform, continuous fibro-protein mat. As demonstrated by the mechanical testing, this structural transformation optimizes energy dissipation without risking compaction or stone-like hardening. No tests have yet been conducted on the wool pellets following repeated wetting and drying cycles, nor have their mechanical properties under wet conditions been assessed. These evaluations are currently underway, together with studies on biodegradability and the long-term stability required for outdoor sports surfaces. The results presented should therefore be regarded as part of an initial study and do not yet provide evidence of long-term durability.

Under field conditions, UV/solar radiation induces photo-oxidative degradation of wool keratin, producing yellow chromophores and reducing fiber mechanical integrity; these photochemical modifications may enhance subsequent soil biodegradation by increasing fiber oxidation, wettability, fragmentation and microbial accessibility [19,45]. Although disinfection is expected to limit bacterial growth, the long-term biological stability of the material, including the potential development of mold and bacteria under real-world field conditions, still requires further assessment.

Unlike synthetic microplastics that have persisted in ecosystems for centuries, the narrow C/N ratio (≈4) [46] ensures that displaced wool particles naturally biodegrade in the surrounding soil without leaving toxic residues.

When contrasted with other natural alternative infills under development, raw wool pellets demonstrate clear technical advantages:

- Comparison with cork: cork-based infills suffer from an extremely low specific gravity, causing them to float during heavy rainfall events and migrate out of the pitch boundaries [47,48]. The mineral-compounded wool pellets (with densities approaching 1.0 g/cm^3^) could overcome this flotation liability.
- Comparison with corn cob: corn-derived granular infills may present a player-safety drawback because granular organic infills [48], including corn kernels, have been described as non-resilient, prone to higher grip, and somewhat abrasive under dry conditions. In addition, corn-cob granulates are angular/irregular particles with reported hardness around Mohs 4.5, features that can increase the potential for abrasive skin contact. Since skin-surface friction and contact with rough/hard counter-surfaces are implicated in abrasion-type injuries during sliding or falls on artificial turf, these properties may increase the risk of friction-induced skin trauma [49]. In contrast, the swollen, soft fibro-protein matrix of wool offers an inherently non-abrasive, high-comfort contact interface.

A recent study compared the physical performance and fatigue responses associated with different plant-based infills with those of SBR and natural grass [48]. No single plant-based infill consistently outperformed the others across all evaluated variables, underscoring the need for a case-by-case assessment. The findings indicate that certain plant-based infills may provide performance characteristics comparable to those of conventional systems. However, their suitability for broader implementation will require further evaluation of their long-term performance, environmental impact, and behavior under different application conditions.

The primary objective of ongoing research is the fine-tuning of the pelletization mechanics to guarantee long-term operational stability. Current efforts are focused on refining the milling processes to compress pellet diameters down to 2.5 mm while simultaneously maximizing volumetric density through advanced mineral salt blending. Additionally, current trials are exploring the encapsulation or modification of the wool pellets with trace bio-based polymers. This surface modification aims to accurately modulate the rate of hydro-interaction and moisture release, enabling optimal mechanical performance and prolonged operational lifespans across diverse climate zones without compromising the inherent biodegradability of the core material.

## 5. Conclusions

This study demonstrates the technical, biomechanical, and environmental feasibility of utilizing raw wool waste pellets as a sustainable, non-toxic infill alternative for synthetic sports turf. Thermogravimetric analysis confirmed that the incorporation of mineral additives successfully modulated the stable inorganic content and optimized volumetric density without destabilizing the compound. Mechanical evaluations under a rigid concrete subbase revealed a quantifiable mass-dependent damping effect, where increasing the raw pellet dosage systematically elevated shock absorption. Crucially, a moisture-induced wet-and-dry cycle triggered pellet de-agglomeration and fiber swelling, permanently modifying the system to achieve compliant vertical deformation and protective energy restitution values. When integrated with a prefabricated polyolefin elastic base, all wool-based infill formulations, including both raw and mineral-loaded configurations, exhibited values within the selected ranges under EN 15330-1, FIFA, and World Rugby regulations, while could also effectively prevent particle flotation during rain events.

Beyond this biomechanical compliance, the hydrophilic structure of the keratin matrix could contribute to hydro-thermal regulation, potentially reducing surface temperature and electrostatic charge accumulation. This hydro-active behavior is projected to drive a reduction in municipal pitch irrigation demands compared to traditional, hydrophobic styrene-butadiene rubber (SBR) crumbs, minimizing the operational carbon footprint of sports complexes. Finally, by leveraging the pre-existing industrial infrastructure of the organic fertilizer sector, the production of these specialized wool pellets can be readily scaled up with minor processing adjustments. This innovative approach provides a compliant, potentially non-abrasive, and biodegradable infill that strategically anticipates the EU 2031 microplastics ban, transforming a costly agricultural liability into a high-value circular economy asset that supports the economic survival of rural sheep farming.

## 6. Patents

This work builds upon previous developmental phases that led to the patent EP24382712.8, titled “Infill for artificial turf” [50].

## Figures and Tables

**Table 1:** Mechanical property requirements for artificial turf surfaces across different regulatory bodies EN 15330-1; 2013, FIFA Test Manual Requirements; Oct 2015, World Rugby 2017.

Parameter	EN 15330-1	FIFA	World Rugby (WR)
Shock absorption (SA) (%)	55–70	55–70	57–68
Vertical deformation (VD) (mm)	4–9	4–11	6–10
Energy restitution (ER)	-	-	22–48

**Table 2:** Physical characteristics, dimensions, and bulk density of the manufactured wool-based infill pellets (Mean values and standard deviations).

Sample No.	Sample Specification	Size (mm Ø)	Bulk Density (g/cm^3^)
1	Clean uncompressed wool	—	0.03 ± 0.08
2	Raw wool pellets (Control)	6.0 ± 0.1	0.39 ± 0.09
3	Raw wool pellets with 25% Dolomite	6.0 ± 0.1	0.67 ± 0.12
4	Raw wool pellets with 50% Dolomite	6.0 ± 0.2	0.90 ± 0.15
5	Raw wool pellets with 50% Barite	6.0 ± 0.2	0.93 ± 0.16
6	Raw wool pellets with 50% Barite	4.5 ± 0.1	0.89 ± 0.11
7	Raw wool pellets with 50% Barite	3.0 ± 0.1	0.87 ± 0.15

**Table 3:** Mass fraction distribution of moisture, organic matter, and inorganic residue determined via TGA (Mean values and standard deviations).

Sample No.	Sample Specification	Water (%)	Organic Fraction (%)	Inorganic Fraction (%)
1	Clean uncompressed wool	9.2 ± 1.2	88.8 ± 1.5	2.0 ± 0.9
2	Raw wool pellets	9.4 ± 0.7	76.6 ± 0.4	14.0 ± 0.8
3	Raw wool pellets with 25% Dolomite	6.2 ± 0.4	61.9 ± 0.5	31.8 ± 1.1
4	Raw wool pellets with 50% Dolomite	4.3 ± 0.1	52.3 ± 0.5	43.4 ± 1.3
5	Raw wool pellets with 50% Barite	5.1 ± 0.6	35.6 ± 0.7	59.2 ± 0.9

**Table 4:** Mechanical properties (SA, VD, and ER) of wool pellet infills evaluated directly on a rigid concrete subbase (Mean values, standard deviations).

Sample	Infill Specification	Shock Absorption (SA) %	Vertical Deformation (VD) mm	Restitution Energy (ER) %
A	Without wool pellets	22.2 ± 0.7	2.5 ± 0.2	62.2 ± 0.9
B	100 g raw wool pellets	31.2 ± 3.2 *	3.5 ± 0.4 *	48.4 ± 4.3 *
C	100 g raw wool pellets and 600 mL water	37.5 ± 1.7 *	4.4 ± 0.1 *	43.6 ± 2.2 *
D	200 g raw wool pellets	44.1 ± 2.4 *	5.4 ± 0.2 *	35.7 ± 3.3 *
E	200 g raw wool pellets and 600 mL water	47.1 ± 3.5 *	5.9 ± 0.7 *	31.2 ± 3.6 *
F	270 g raw wool pellets (25% dolomite) and 300 mL water	39.2 ± 3.9 *	4.2 ± 0.6 *	39.3 ± 4.4 *
G	400 g of raw wool pellets (50% dolomite) and 300 mL water	39.4 ± 2.5 *	4.2 ± 0.2 *	36.7 ± 3.1 *

* Indicates significant differences compared to sample A, *p* < 0.05.

**Table 5:** Mechanical properties (SA, VD, and ER) of wool pellet infills evaluated over a 23 mm ProPlay elastic shockpad (Mean values, standard deviations).

Sample	Sample Specification	Shock Absorption (SA) %	Vertical Deformation (VD) mm	Restitution Energy (ER) %
A	Without wool pellets	60.8 ± 1.2	7.5 ± 0.3	36.5 ± 1.7
B	100 g raw wool pellets	62.2 ± 0.6	8.0 ± 0.0 *	35.1 ± 0.6
C	100 g raw wool pellets and 600 mL water	64.3 ± 1.1 *	8.4 ± 0.1 *	32.0 ± 1.4 *
D	200 g raw wool pellets	63.1 ± 0.7 *	8.4 ± 0.2 *	32.7 ± 1.4 *
E	200 g raw wool pellets and 600 mL water	66.7 ± 0.9 *	8.9 ± 0.2 *	29.3 ± 1.5 *
F	270 g raw wool pellets (25% dolomite) and 300 mL water	66.6 ± 1.1 *	9.0 ± 0.5 *	29.7 ± 1.7 *
G	400 g of raw wool pellets (50% dolomite) and 300 mL water	65.2 ± 1.6 *	8.5 ± 0.1 *	32.1 ± 1.1 *

* Indicates significant differences compared to sample A, *p* < 0.05.

## Data Availability

The original contributions presented in this study are included in the article. Further inquiries can be directed to the corresponding author.
